# Plasmacytoma of the Skull-base: A Rare Tumor

**DOI:** 10.7759/cureus.2073

**Published:** 2018-01-15

**Authors:** Abhilasha Siyag, Tej P Soni, Anil K Gupta, Lalit M Sharma, Naresh Jakhotia, Shantanu Sharma

**Affiliations:** 1 Radiation Oncology, Bhagwan Mahaveer Cancer Hospital and Research Centre, Jaipur; 2 Surgical Oncology, Bhagwan Mahaveer Cancer Hospital and Research Centre, Jaipur; 3 Medical Oncology, Bhagwan Mahaveer Cancer Hospital and Research Centre, Jaipur; 4 Radiation Oncology, Sawai Man Singh Medical College and Hospital, Jaipur

**Keywords:** plasmocytoma, skull-base lesion

## Abstract

Plasmacytoma of the skull-base is a rare entity. Differential diagnosis includes chordoma, osteosarcoma, carcinoma nasopharynx, meningioma, metastatic carcinoma, lymphoma, and multiple myeloma. Accurate and precise diagnosis is extremely important for plasmacytoma of the skull-base as its treatment and prognosis is different from other skull-base lesions. A 41-year-old man presented with concerns of headache, diplopia, and left eye strabismus. A magnetic resonance image (MRI) of his brain showed a large expansile mass measuring 51 mm involving the clivus and central skull-base. Trans-sphenoidal tumor decompression was done. A biopsy confirmed the plasmacytoma. A positron emission tomography-computed tomography (PET-CT) scan showed a single 2-(18F) fluoro-D-glucose (FDG) avid lesion at the skull-base. The results of all other relevant investigations such as hemoglobin, renal function test, serum calcium, serum protein immunoelectrophoresis, serum quantitative immunoglobulin, bone marrow biopsy, serum lactate dehydrogenase, and beta-2 microglobulin levels were within normal limits. He was treated with radical radiotherapy. He developed complete clinical response after radiotherapy.

## Introduction

Solitary plasmacytoma is characterized by the localized proliferation of neoplastic monoclonal plasma cells. It is a rare tumor and makes less than 10% of all plasma cell neoplasms [1]. It is subdivided into two distinct forms, extramedullary plasmacytoma and solitary bone plasmacytoma, distinguishable by the sites of origin. Vertebra and skull bones are the most commonly affected bones for solitary bone plasmacytoma [1]. Extramedullary plasmacytoma commonly arises from the head and neck region, nasal cavity, and nasopharynx [1]. Plasmacytoma originating from the skull-base is an extremely rare tumor, and very few cases have been described in the literature [2].

## Case presentation

**Figure 1:**
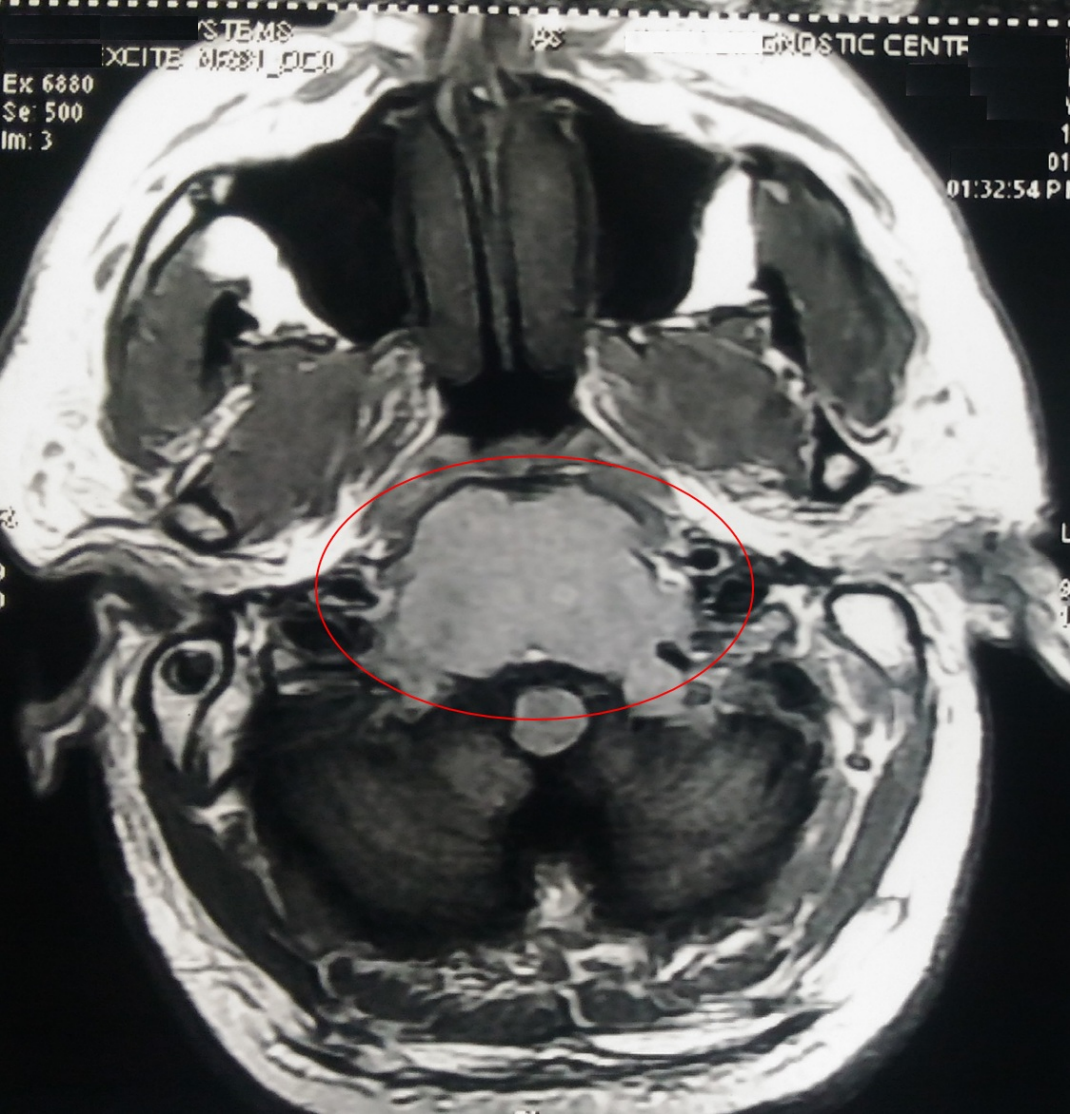
MRI showing a large heterogenous expansile lesion measuring 51 mm x 50 mm involving the central skull base, clivus with erosion of adjacent bones, and partially encasing bilateral ICA ICA: internal carotid artery; MRI: magnetic resonance image.

A 41-year-old man presented with concerns of headache, diplopia, and left eye strabismus lasting two months. There was no history of nausea, vomiting, and seizure. He had no other medical conditions such as hypertension, diabetes, cardiovascular disease or stroke. A neurological examination showed left eye sixth cranial nerve palsy. There was no sensory or motor deficit in his upper and lower limbs. A magnetic resonance image (MRI) of the brain showed a large heterogeneous, expansile lesion measuring 51 mm x 50 mm, involving the central skull-base and clivus with the erosion of adjacent bones, partially encasing the bilateral internal carotid artery (ICA) suggestive of chordoma (Figure 1). 

**Figure 2:**
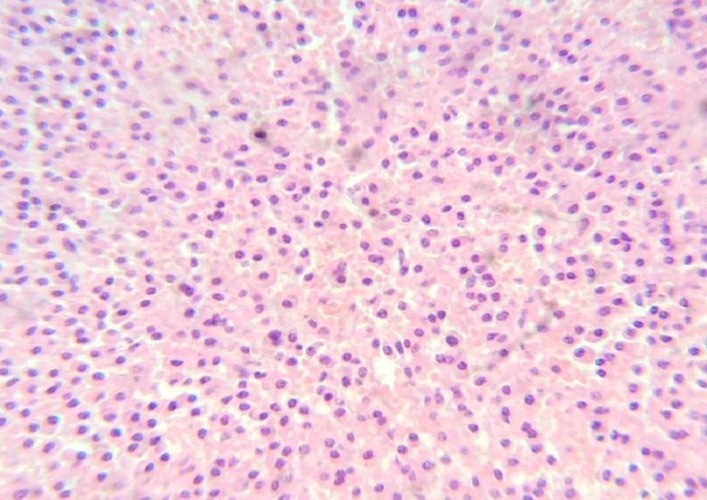
40x view of hematoxylin and eosin (H&E) stained section showing proliferation of the plasma cells with moderately abundant cytoplasm, suggestive of plasma cell neoplasm

Trans-sphenoidal decompression of the tumor was done by a team of neurosurgeons and head-and-neck surgeons. Biopsy from the lesion showed proliferation of the plasma cells with moderately abundant cytoplasm and was reported as plasma cell neoplasm (Figure 2).

**Figure 3:**
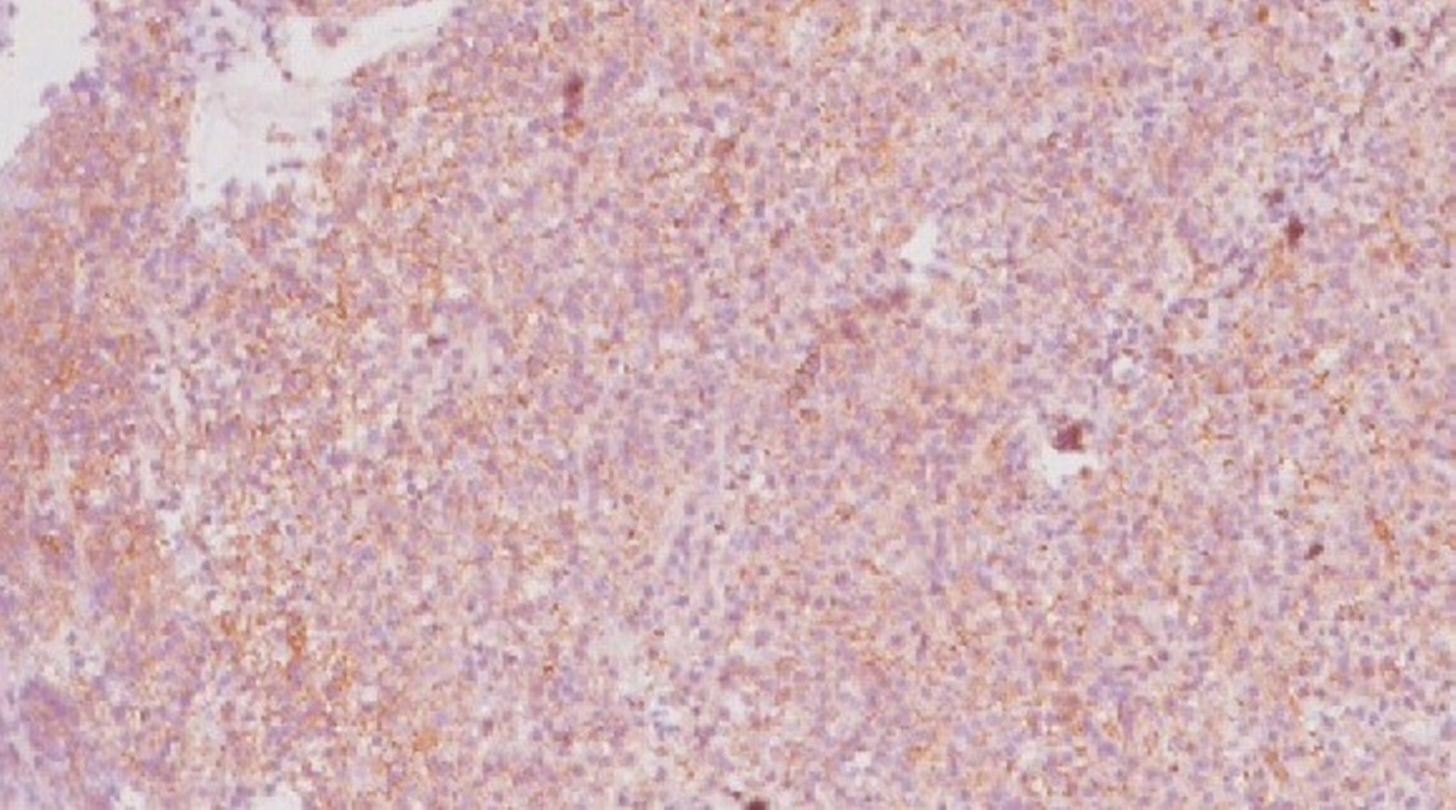
Plasma cells are highlighted by immunohistochemical staining for CD138 (immunohistochemistry, 10x view)

Immunohistochemistry analysis showed CD-138 positive, CD-38 positive, CD-56 positive, and CD-45 negative cells (Figure 3).

**Figure 4:**
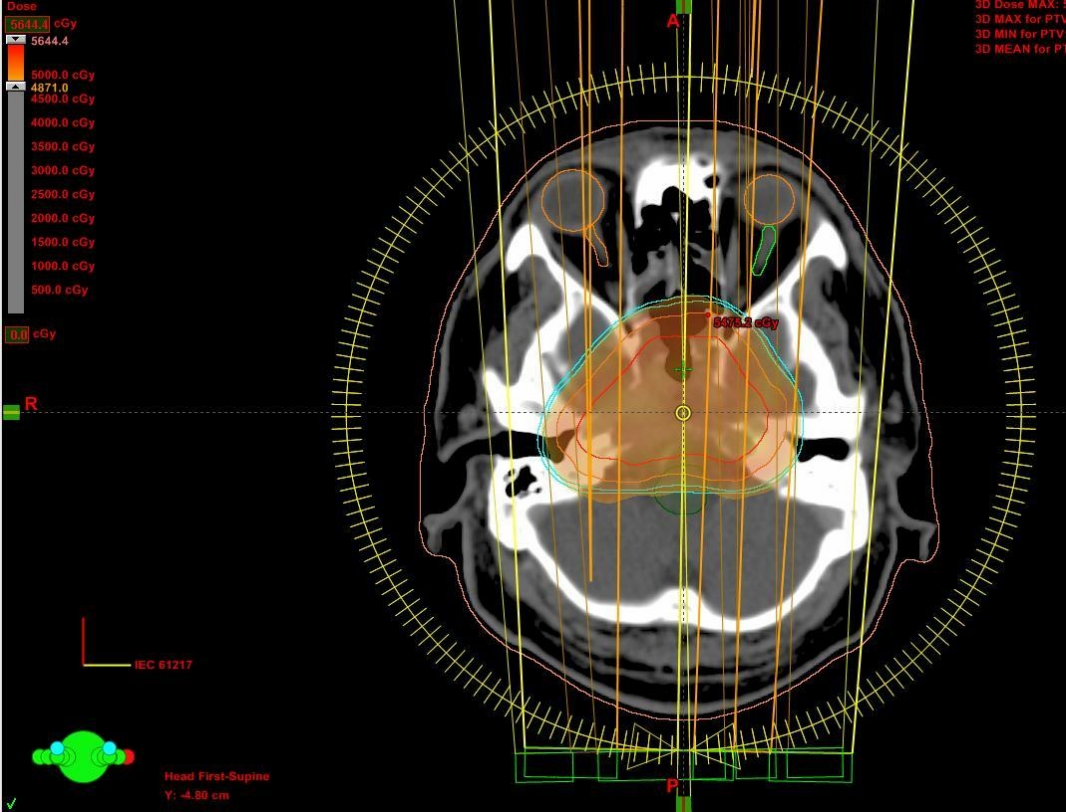
Axial planning computed tomography slice showing typical dose color-wash and dose distribution of intensity modulated radiotherapy by the RapidArc technique

The skeletal survey, bone marrow biopsy, serum protein immunoelectrophoresis (SPEP), serum quantitative immunoglobulins levels, serum lactate dehydrogenase, calcium, albumin, renal function test, and beta-2 microglobulin levels were within normal limits. Bence Jones protein in the 24-hour urine sample was absent. The positron emission tomography-computed tomography (PET-CT) scan showed a single 2-(18F) fluoro-D-glucose (FDG) avid lesion at the base of the skull as described in the MRI of the brain. He was treated with radical radiotherapy with intensity modulated radiotherapy technique to a dose of 50 Gy in 25 fractions over a five-week duration by 6-megavolt (MV) x-rays on a modern linear accelerator (Figure 4).

**Figure 5:**
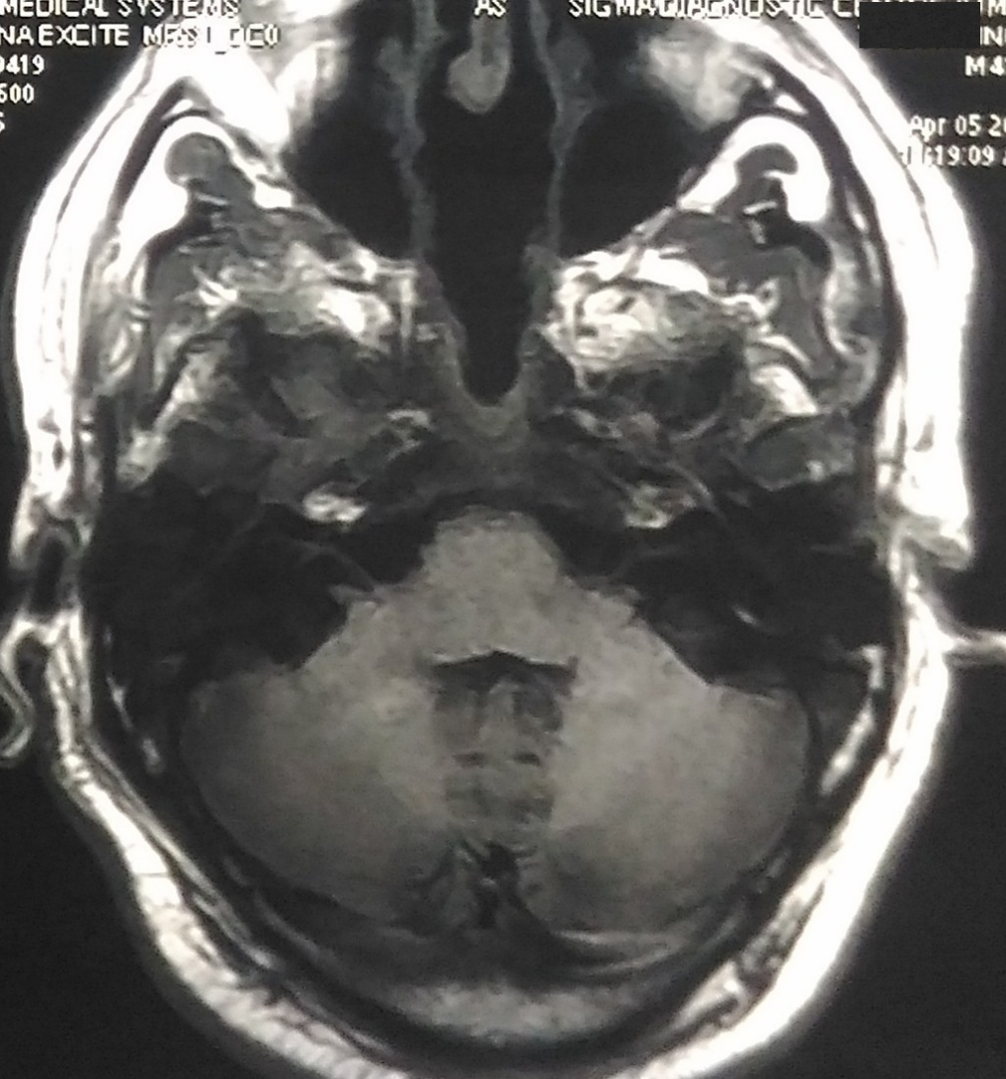
MRI after three months of radiotherapy showing complete remission of the skull-base plasmacytoma lesion MRI: magnetic resonance image.

After radiotherapy, he got complete symptomatic relief from headache and diplopia. A second MRI scan to assess the response following three months of radiotherapy showed complete remission of the lesion (Figure 5). 

One year after radiotherapy, investigations including PET-CT scan, MRI scan, serum quantitative immunoglobulins level, SPEP, and beta-2 microglobulin level were within normal limits with no signs of the recurrence.

## Discussion

Plasmacytoma of the skull-base is a very rare disease entity [3]. The radiological differential diagnosis of the base of skull plasmacytoma includes carcinoma nasopharynx, chordoma, meningioma, osteosarcoma, lymphoma, pituitary adenoma, metastatic carcinoma, eosinophilic granuloma, and multiple myeloma [3]. It usually presents with headache, diplopia, and visual deficit [3]. Direct compression or involvement of the cranial nerves causes neuropathy and neurologic symptoms. Most commonly, the sixth cranial nerve (abducens nerve palsy) is involved, followed by the second, fifth, seventh, and eighth cranial nerves [3]. Multiple myeloma and plasmacytoma are two ends of the same disease-spectrum characterized by the malignant proliferation of plasma cells. Findings such as a solitary lesion, bone marrow biopsy with less than 5% plasma cells, monoclonal protein (M protein) level less than 2g/dL, the absence of Bence Jones proteins in the urine, no anemia or hypercalcemia, and normal renal function tests support the diagnosis of plasmacytoma [4]. Multiple myeloma characteristics are more than 10% plasma cells in bone marrow biopsy, M protein level more than 3g/dL, multiple lytic bone lesions, hypercalcemia, renal failure, and anemia [5]. Complete and thorough workup to rule out multiple myeloma is necessary before the local treatment of plasmacytoma.

Trans-sphenoidal surgery is required for diagnosis and tumor debulking in patients with neurologic deficits due to large tumor mass effect or in recurrent or residual lesions [3]. Radiotherapy is the treatment of choice for the solitary plasmacytoma of the skull-base as it is a radiosensitive tumor [3]. Radiotherapy to the dose of 50 Gy in five weeks has excellent outcomes with local control rates of more than 85% [3]. Adjuvant chemotherapy for the solitary bone plasmacytoma has no proven beneficial role or impact on the progression-free survival or progression to multiple myeloma [4]. Despite the full treatment, 50% to 60% of solitary plasmacytoma patients progress to multiple myeloma, as the systemic failure, within a median time interval of two years [6]. High conversion rates to multiple myeloma warrant regular follow up after radiotherapy.

## Conclusions

An accurate and precise diagnosis is extremely important for plasmacytoma of the skull-base as its treatment and prognosis is different from other skull-base lesions. Radiotherapy is the treatment of choice for the solitary plasmacytoma of the skull-base as it is a radiosensitive tumor.
